# A Methodology for Predicting the Phase Fraction and Microhardness of Welded Joints Using Integrated Models

**DOI:** 10.3390/ma16072599

**Published:** 2023-03-24

**Authors:** Ji-Hyo Song, Kyung-Woo Yi

**Affiliations:** Department of Materials Science and Engineering, Seoul National University, Seoul 08826, Republic of Korea; songjoe@snu.ac.kr

**Keywords:** heat-affected zone, grain growth model, prior austenite grains, continuous cooling transformation, phase fraction, microhardness

## Abstract

Understanding the phase transformation and fraction affected by thermal changes is imperative for ensuring the safety of a welded joint. This study proposes a methodology for predicting the phase transformation and fraction of a welded joint using an integrated model. The integrated model includes a heat transfer model and procedures for predicting phase fraction and microhardness. The heat transfer model was developed to simulate the heat transfer in a welded joint and obtain the thermal cycles. The procedure consists of obtaining the peak temperature, austenite fraction, prior austenite grain size (PAGS), and t_8/5_ (the cooling time between 800 and 500 °C). A database was constructed based on the continuous cooling transformation (CCT) diagram using PAGS and t_8/5_ as the variables. The phase fraction was then predicted by considering the PAGS with t_8/5_ from the database. The predicted phase fraction and microhardness were in good agreement with those determined experimentally, demonstrating the reliability of the methodology. This methodology provides a more realistic understanding of phase transformation and facilitates the prediction of the phase fraction and microhardness under various welding conditions that have experimental limitations.

## 1. Introduction

Owing to societal pressures, there is a growing demand for safer welding processes in various fields, including the aerospace, automotive, shipbuilding, and construction industries. Studies are being actively conducted to analyze welding safety and the causes of deterioration in welded joints [1,2].

The heat-affected zone (HAZ) of a welded joint is the most important region influencing its safety, and several studies have focused on evaluating the characteristics of the HAZ. The microstructures in the HAZ and fusion zone (FZ) are different from those in the base metal (BM), and the transformations of these microstructures cause changes in mechanical properties as well as welding cracks, which significantly decrease welding safety [3,4]. The coarse grains and formation of martensite cause a reduction in toughness, and martensite–austenite (M/A) mixtures act as crack initiation sites, producing local brittle zones (LBZs) [5,6,7]. Therefore, predicting the phase transformation in a welded joint during the welding process is necessary to maximize welding safety.

Different thermal cycles are produced in welded joints owing to differences in heat transfer, thereby generating microstructures with varying phase distributions and mechanical properties. Phase transformations during the welding process are very complex, and analyzing the microstructures is difficult because they must be observed in a very narrow area. Thus, developing a method for understanding the phase transformations, predicting the microstructure, and characterizing the mechanical properties is necessary.

Numerous studies have been conducted to analyze the characteristics of welded joints. Shi et al. investigated the microstructure and mechanical properties of a Q690E steel after underwater dry welding and underwater local cavity welding [8]. They experimentally compared the microstructure and mechanical properties of welded joints according to the welding process. Wang et al. applied fiber laser to weld a nano-scale precipitate-strengthened C–Mn steel (NPS steel) and studied the effect of welding speed on the microstructure, microhardness, and yield strength of the welded joint [9]. 

Few studies have used a thermal simulator to reproduce a welding thermal cycle and analyzed the characteristics of welded joints [10,11]. Wu et al. studied the microstructure and microhardness of a simulated HAZ subjected to various peak temperatures and established the continuous cooling transformation (CCT) diagram of the coarse-grained HAZ (CGHAZ) for a peak temperature of 1350 °C [11]. Di et al. simulated inter-critical HAZ (ICHAZ), which is known as the weak area of a weld, and studied the relationship between the microstructure and toughness for peak temperatures of 750, 780, and 800 °C [12]. To analyze the microstructure and physical properties of a welded joint at a desired location or welding condition, experimental studies are appropriate. However, numerous experiments are necessary to predict the changes in the entire welded joint, because the thermal cycles experienced by it are location dependent, which results in differences in microstructure and mechanical properties.

CCT diagrams are useful for predicting the microstructure of welded joints because they provide information about the phase transformation during cooling. Numerous studies have been conducted to predict phase fraction using CCT diagrams [12,13,14,15]. Yang et al. proposed a model that combines experiments and numerical simulations to predict the microstructure and microhardness of the HAZ [12]. A thermal simulator reproduces a thermal cycle with the same peak temperature but different cooling rates. The expansion of the sample is recorded by a dilatometer to determine the phase transformation temperatures, and these results are used to obtain the CCT diagram. The phase fraction is predicted by applying the cooling rate obtained via numerical simulation to the CCT diagram. Zong et al. used a thermal simulator and dilatometry to obtain a CCT diagram [13]. Using this CCT diagram, the difference in microstructure according to the difference in the cooling rate at a specific peak temperature was analyzed. Deng used two CCT diagrams in a welded joint by considering different prior austenite grain sizes (PAGSs) [14]. Zhang et al. also used a dilatometry to attain a CCT diagram and analyzed the difference in phase transformation temperature and behavior during welding according to the difference between low-alloyed and high-alloyed steel [15].

The initial phase transformation of the welding material usually occurs at the austenite grain boundaries during the cooling process. This suggests that PAGS is a crucial factor in the kinetics of phase transformation [16,17,18]. The PAGS, combined with the cooling rate, creates a variance in the phase transformation and the microstructure that forms after the cooling process. This means that the PAGS acts as an important variable in the CCT diagram, which displays the phase-transformation temperature. Therefore, the PAGS must be predicted to evaluate the correct CCT diagram and predict the phase fraction by accurately simulating the phase transformation.

Schönmaier et al. recognized the difference in PAGS with respect to the location and applied three CCT diagrams with varying peak temperatures to realistically simulate the thermal cycles of various regions of a HAZ [19]. Although CCT diagrams were thoroughly used, they evaluated the CCT diagrams by performing dilatometry experiments and microstructure and microhardness analysis, which can be time consuming. Previous studies have used CCT diagrams obtained via simulations instead of experiments to overcome this limitation [20,21,22,23]. In these studies, a single predict CCT diagram was used to predict the phase fraction in the entire welded joint without considering the differences in PAGS with location.

In this study, a methodology for predicting the phase fraction and microhardness of the entire welded joints is presented, which is a combination of a heat transfer model and a procedure to obtain the peak temperature, austenite fraction, PAGS, and t_8/5_ (the cooling time between 800 and 500 °C). In addition, a database was constructed based on a CCT diagram calculated using the composition and PAGS (applying the cooling rate to this diagram). Finally, the fractions of the martensite, bainite, ferrite, and perlite phases were predicted by considering PAGS, which was predicted using a grain growth model with t_8/5_. This methodology was verified by comparing its results with those of experiments. Then, it was used for analyzing the phase transformation during welding and for evaluating microhardness as a function of the phase fraction.

## 2. Modeling Methodology

### 2.1. Integrated Model for Predicting the Phase Fraction and Microhardness of Welded Joints

**Figure 1:**
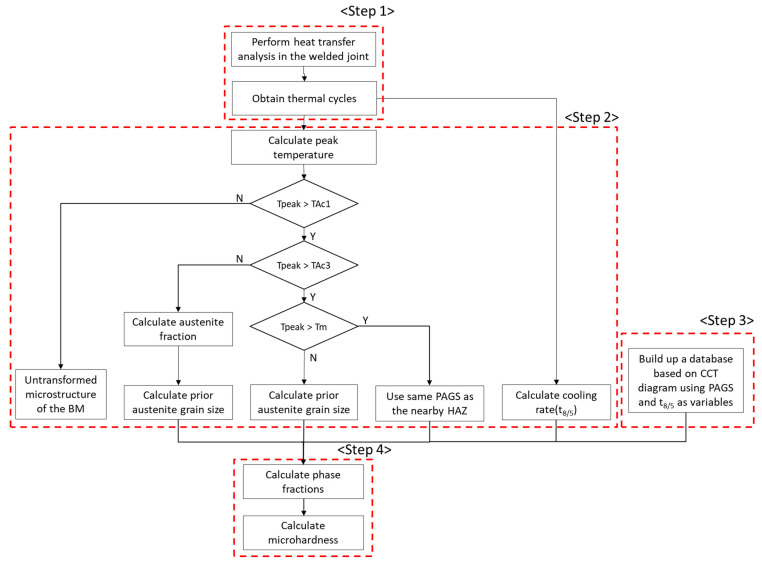
Flowchart for predicting the phase fraction and microhardness in welded joint.

Figure 1 displays a flowchart of the new integrated model used to predict the phase fraction and microhardness of welded joints. The model consists of the following steps: Step 1: A heat transfer analysis is conducted on the welded joint to obtain the thermal cycles. Step 2: The peak temperature, austenite fraction, PAGS, and t_8/5_ are predicted for each location being analyzed using the results obtained in Step 1. Step 3: A database is created based on a CCT diagram calculated according to the composition with PAGS and t_8/5_ as variables. Step 4: The phase fraction and microhardness are predicted by combining the database created in Step 3 with the PAGS and t_8/5_ obtained from Step 2.

The heat transfer model was used to analyze the heat transferred through the welded joint and obtain the thermal cycles, as described in Step 1 of the flowchart (Figure 1). The thermal cycles were used in Step 2 to predict the t_8/5_ required in Step 4. In Step 2, the austenite fractions (with peak temperature being the only variable affecting austenite transformation), PAGS, and t_8/5_ were predicted for each location of the welded joint. Austenite transformation started when the temperature reached Ac_1_ and ended when the temperature reached Ac_3_. The PAGS can be measured experimentally; however, the method is complicated and inefficient. Therefore, the use of a grain growth model for predicting PAGS is reasonable. The value of t_8/5_ was obtained from the cooling rate, which was evaluated by decreasing the temperature from 800 to 500 °C. Steps 1 and 2 were coupled procedures, whereas Step 3 was independent. The values of PAGS and t_8/5_ varied with the location of the welded joint. Moreover, for a given location, the values of PAGS and t_8/5_ varied with the welding conditions. This prediction implies that the CCT diagram must be reevaluated each time the composition and PAGS vary. An alternative is to prepare a database containing the composition, PAGS, and t_8/5_. The phase fraction can then be predicted directly from the values of PAGS and t_8/5_ listed in the database without the need for predicting a CCT diagram. Finally, Step 4 involved predicting the phase fraction and microhardness. The phase fraction was predicted using the PAGS and t_8/5_ values from the database. Because no CCT diagram was available for the FZ, the PAGS in the FZ was assumed to be the same as that of the HAZ adjacent to it. In other words, the CCT diagram of the HAZ was assumed to be the CCT diagram of the FZ [14]. The microhardness was predicted by summing the microhardness values of each of the phases weighted by their phase fractions.

### 2.2. Heat Transfer Analysis

In this study, the ANSYS^®^ FLUENT18.2 (Ansys lnc., Pennsylvania, USA) program was used to solve the governing differential equations required to analyze heat transfer in the welded joint. The properties of the material used herein are presented in Table 1. The temperature-dependent density, thermal conductivity and viscosity of case 1 were calculated based on the composition in Table 2 using JMatPro^®^13 (Sente software co., Ltd, Guildford, UK) while the density, thermal conductivity, and viscosity of case 2 were obtained from the literature [24]. 

The heat source model employed in this study was a volumetric heat flux with a constant heat density, determined by welding current, voltage, velocity, and heat efficiency [25,26]. The welding conditions used for numerical simulation are presented in Table 3. The heat in the welded joint was transferred via conduction and convection of the fluid flow in the FZ. A heat transfer model was used in this study to account for the fluid flow in the FZ, as many studies have concluded that the convective heat transfer caused by fluid flow significantly influences temperature distributions and thermal cycles [27,28,29,30]. An ambient environment was considered, and heat transfer occurred through the surface of the weld material via convection and radiation. The small area where a BM and filler metal (FM) mix is referred to as the FZ. Considering the insignificant size of the FZ, the properties of the BM were considered to represent the entire welding material in this study.

The governing equations describing the heat transfer include the continuity (Equation (1)), momentum (Equation (2)), and energy conservation (Equation (3)) as well as the standard k-epsilon turbulence, which was used to account for the effects of fluid flow. The governing equations are displayed below.

The continuity equation is

(1) $$\frac{\partial \rho }{\partial t}+\nabla \cdot \left(\rho \vec{v}\right)=0,$$

where $\vec{v}$ is the fluid velocity, and ρ is the density.

The momentum equation is

(2) $$\frac{\partial }{\partial t}\left(\rho \vec{v}\right)+\nabla \cdot \left(\rho \vec{v}\vec{v}\right)=-\nabla P+\nabla \cdot [\mu (\nabla \vec{v}+\nabla {\vec{v}}^{T})]+\rho \vec{g}+\vec{F},$$

where P is the pressure, μ is the viscosity, $\vec{g}$ is the acceleration due to gravity, and $\vec{F}$ is the surface tension or force.

The energy equation is

(3) $$\frac{\partial }{\partial t}\left(\rho H\right)+\nabla \cdot \left(\rho \vec{v}H\right)=-\nabla \cdot \left(k\nabla T\right)+S,$$

where *H* is the enthalpy of the materials, and *k* is the thermal conductivity.

An enthalpy porosity model was used to simulate the solidification and melting behaviors in the welding process. This model accounts for the fluid flow in the solid–liquid phase by assuming a porous region, the porosity of which continuously varies from 1 to 0. This number represents the liquid fraction at each position as the melting or solidification progresses.

The enthalpy (H) equations are

(4) $$\nabla \cdot \left(\rho \vec{v}\right)\frac{\partial }{\partial t}\left(\rho \vec{v}\right)+H=h+\Delta H,\;\Delta H=\beta L,\;h=h_{ref}+\overset{T}{\underset{T_{ref}}{\int }}C_{p}dT,\;\beta =0\;\mathrm{if}\;T<T_{solidus},\;\beta =1\;\mathrm{if}\;T>T_{liquidus},\;\beta =\frac{T-T_{solidus}}{T_{liquidus}-T_{solidus}}\;\mathrm{if}\;T_{liquidus}\leq T\leq T_{solidus},$$

where *L* is the latent heat of the materials, *h* is the sensible enthalpy, and β is the liquid fraction (which is a function of temperature).

In this study, weld heat transfer analysis was performed by considering two welding methods, SAW and GTAW, although the partial analysis was performed on both types of welding, while full analysis was only performed on the GTAW. 

### 2.3. Prediction of Austenite Fraction and Prior Austenite Grain Size

During the heating process, austenite begins to form at Ac_1_ (697 °C) and the transformation to austenite is complete at Ac_3_ (817 °C). The austenite fraction increases linearly from Ac_1_ to Ac_3_. For example, half of the BM microstructure is austenitized if the peak temperature is 757 °C (exactly halfway between Ac_1_ and Ac_3_).

Austenite grows at temperatures at or above Ac_3_, with different thermal cycles occurring in each location producing a range of PAGSs distributed throughout the welded joint. Isothermal grain growth models that account for the composition of the metal have been developed [31,32,33]. These models can be extended to predict non-isothermal grain growth using the rule of additivity. The PAGS was predicted using the following equation:

(5) $$D^{1/n}-D_{0}^{1/n}=K\int_{t1}^{t2}\exp(-\frac{Q_{app}}{RT\left(t\right)})dt,$$

where *D* is the final grain size, *D*_0_ is the initial grain size, *n* is the time exponent, *K* is the kinetic constant, *Q_app_* is the activation energy for the grain boundary movement as a function of composition, *t* is the time, and *T*(*t*) is the time-dependent thermal cycle. The variables used in the integral, *t*_1_ and *t*_2_, represent the grain growth start and end times, respectively, as well as the time spent at temperatures above Ac_3_. The times *t*_1_, *t*_2_, and *T*(*t*) were obtained from the simulated results, and the initial grain size was the grain size before the growth of austenite, which was measured as 5 μm in this study. The time exponent, kinetic constant, and activation energy were taken from [31]. The t_8/5_ was predicted in Step 2 from the thermal cycles, which was used for the phase fraction analysis in Step 4 together with the PAGS. 

### 2.4. Prediction of Microhardness

The microhardness was predominantly affected by the microstructure, making it useful for evaluating the accuracy of the predicted phase fractions. The microhardness was predicted using the rule of mixtures, which is expressed as follows:

(6) $$HV=X_{M}\times HV_{M}+X_{B}\times HV_{B}+(X_{F}+X_{P})HV_{F+P},$$

where *HV* is the total hardness; *X_M_*, *X_B_*, *X_F_*, and *X_P_* are the volume fractions of martensite, bainite, ferrite, and pearlite, respectively, and *HV_M_*, *HV_B_*, and *HV_F+P_* are the hardness of martensite, bainite, and a mixture of ferrite and pearlite, respectively. The microhardness values of the phases were predicted using empirical formulas that accounted for the chemical composition and cooling rate [34], which are expressed as

(7) $$HV_{M}=127+949C+27Si+11Mn+8Ni+16Cr+21\mathrm{logV},$$

(8) $$\;\;\;\;\;\;\;\;\;HV_{B}=-323+185C+330Si+153Mn+65Ni+144Cr+191Mo\;+logV\left(89+53C-55Si-22Mn-10Ni-20Cr-33Mo\right),$$

(9) $$\;\;\;\;\;\;HV_{F+P}=42+223C+53Si+30Mn+12.6Ni+7Cr+19Mo+logV(10\;-19Si+4Ni+8Cr+130V),$$

where *HV_M_*, *HV_B_*, and *HV_F+P_* are the same as those defined previously for Equation (6), where the compositions are given in terms of the weight percent from the values in Table 2, and V is the cooling rate at 700 °C, which can be obtained from the heat transfer model. The microhardness of each phase was predicted separately considering the compositional difference between the FM and BM. The microhardness of each phase was predicted at 0.1 mm intervals to observe the change caused by the difference in the cooling rate at 700 °C depending on the location and phase [35].

## 3. Experiment

**Figure 2:**
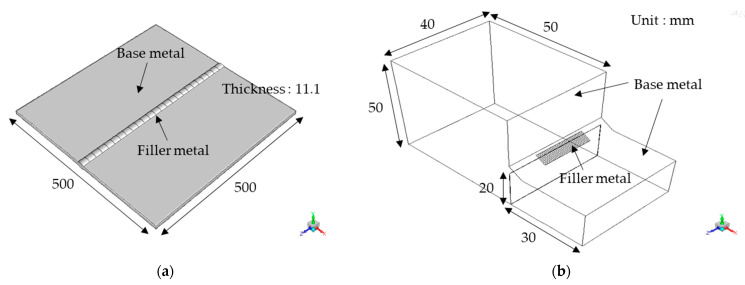
Schematic model geometry for (**a**) SAW and (**b**) GTAW.

Two welding methods were used in this study to weld a BM with an FM: in case 1, SAW was used on ASTM A516-60 and API 2H-50, and in case 2, GTAW was used on SS400 and ER70S-6 steels. The chemical compositions of the materials and welding conditions are listed in Table 2 and Table 3. The chemical compositions were determined using inductively coupled plasma-atomic emission spectrometry (ICP–AES, model: OPTIMA 8300, Perkin-Elmer). Schematics of the welding geometry are shown in Figure 2.

For case 1, a plate with a butt joint with dimensions of 500 mm × 500 mm × 11.1 mm was welded using SAW, and internal and external welding was successively performed at initial temperatures of 27 and 70 °C, respectively. ASTM A516-60 was used as the base metal, while API 2H 50 was used as the filler metal. Because the welded joint was covered with flux during the SAW process, the welded surface was assumed to be covered with 20 mm of flux, and OK Flux 10.72 was used as the flux.

**Figure 3:**
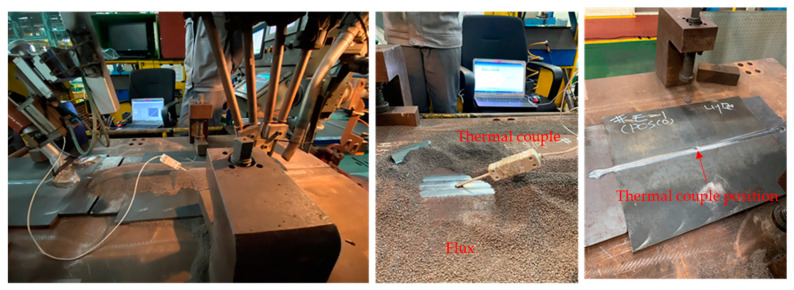
Thermocouple location for measuring thermal cycle during welding process.

A method commonly used for verifying the welding heat transfer model is to compare the thermal cycle or weld bead morphology via experiments and simulations [12,21,36,37]. To evaluate the accuracy of the heat transfer model for case 1, the experimentally obtained thermal cycle and weld bead morphology were compared with those from simulations. The thermal cycles were measured using a thermocouple located at 250 mm in the center of the plate during the internal and external SAW processes, as shown in Figure 3.

For case 2, GTAW was used to weld specimens with dimensions of 50 mm × 50 mm × 40 mm and 30 mm × 20 mm × 40 mm. The initial temperature of the weld specimens prior to welding was 24 °C and argon shielding gas was supplied at flow rate of 15 L/min. A fillet-welded joint was adopted as the weld shape, and the horizontal and vertical leg lengths were 5.4 and 4.4 mm, respectively (see Figure 2). After completion of the single-pass GTAW process, the sizes of the specimens (including those of the FZ, HAZ, and BM) were reduced to 20 mm × 20 mm × 4 mm. SS400 was used as the base metal, while ER70S-6 with a diameter of 2.4 mm was used as the filler metal.

**Figure 4:**
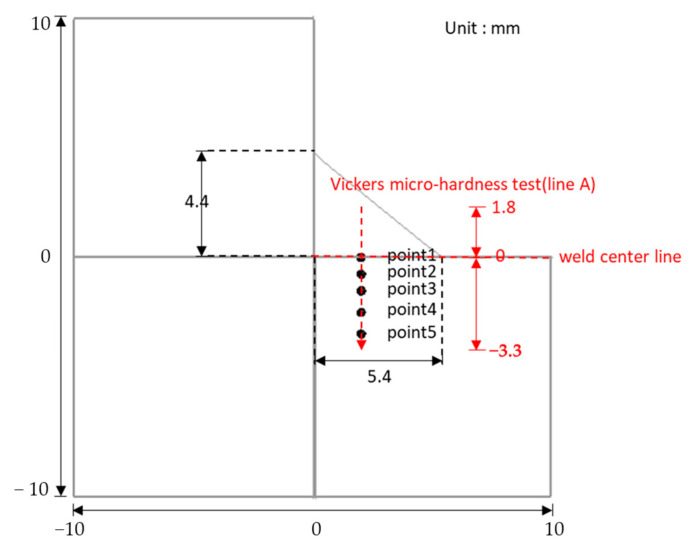
Schematic of welded joint plane and SEM, EBSD analysis positions, and Vickers micro-hardness test line.

**Figure 5:**
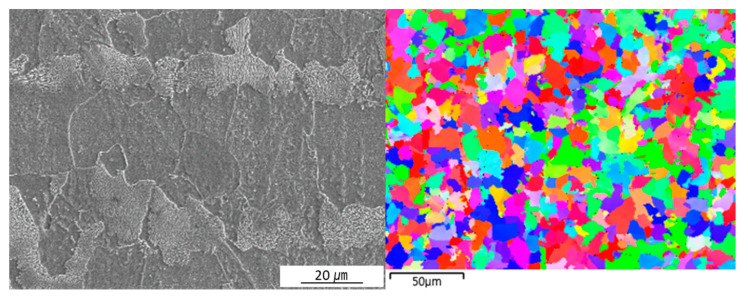
Microstructure and grain morphology of the base metal.

The microstructure and grain morphology of the welded joint were investigated using field emission scanning electron microscopy (FE-SEM, model; SU70, Hitachi, Tokyo, Japan) and electron back-scattered diffraction (EBSD, TSL). These investigations were conducted at five different points of the welded joint along line A shown in Figure 4. The SEM, EBSD, and hardness analyses were conducted along the points and lines shown in Figure 4, and the results were compared with the predicted results at similar locations. The specimen for SEM analysis was polished using SiC paper and diamond suspension, and it was subsequently etched with a 3% Nital solution. The specimens used for the EBSD analysis were prepared by polishing them with SiC paper, diamond suspension, and colloidal silica solution. The microhardness values of the cross sections of the specimens were measured using a Vickers hardness tester (model; HM-122, Mitutoyo, Tokyo, Japan) at intervals of 0.1 mm along the lines shown in Figure 4. The microstructure of the BM for case 2 contained ferrite and pearlite with an average grain diameter of 12 μm, as shown in Figure 5.

## 4. Results and Discussion

### 4.1. Heat Transfer Model Validation for SAW and GTAW

**Figure 6:**
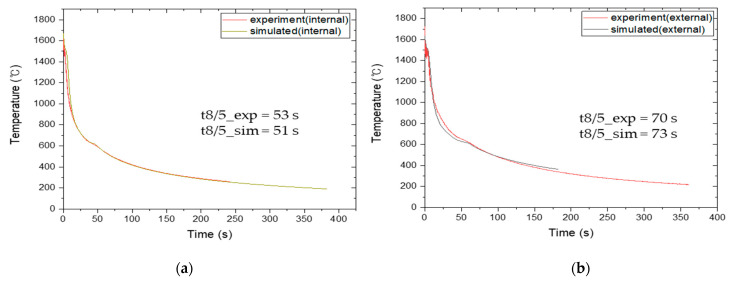
Comparison of experiment and simulated results: (**a**) SAW internal pass and (**b**) SAW external pass.

**Figure 7:**
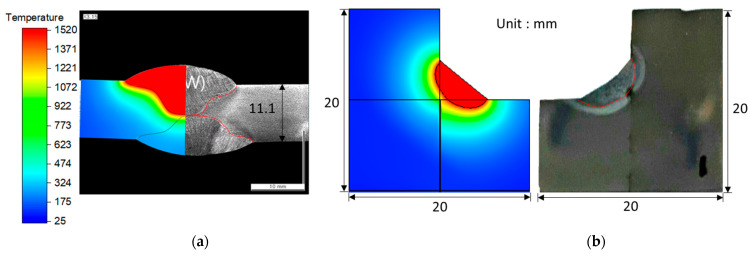
Comparison of weld bead morphology between the experiment and simulated result: (**a**) SAW and (**b**) GTAW.

Figure 6 compares the simulated and experimental thermal cycles and t_8/5_ of the SAW process. The simulated thermal cycles were consistent with the experimental observations. Moreover, the difference between the simulated and measured t_8/5_ was less than 5%, which confirms that the predicted value of t_8/5_ is correct. Figure 7a presents a comparison of the simulated and experimentally obtained cross sections of the weld bead morphology. The black line in the weld cross section was obtained from the numerical analysis, and the red line superimposed on the actual specimen indicates the FZ. The predicted weld bead geometry was in good agreement with the experimental results, which suggests that the validity of the heat transfer model can be verified by comparing the predicted and experimentally obtained cross sections of the weld bead morphology.

Figure 7b compares the simulated and experimentally obtained cross sections of the weld bead morphology corresponding to case 2. Moreover, the simulated results agree with the experimental results.

### 4.2. Thermal Behavior during GTAW 

**Figure 8:**
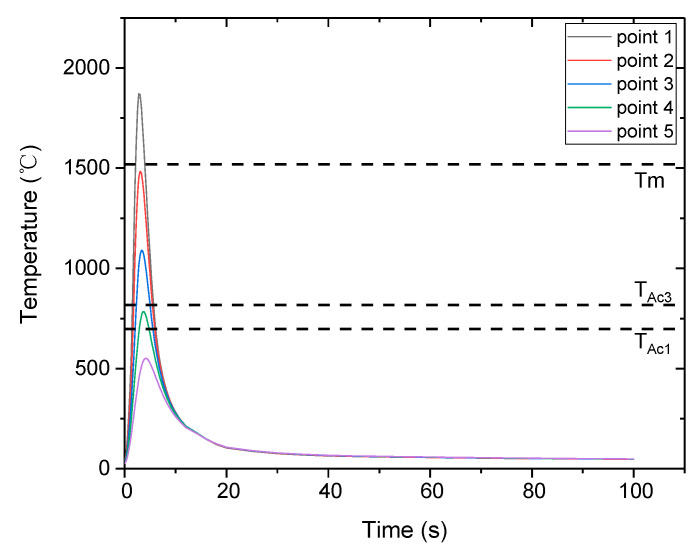
Thermal cycles at different locations.

**Figure 9:**
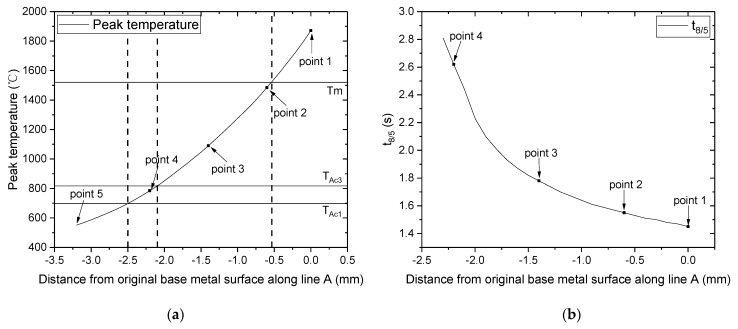
(**a**) Peak temperature and (**b**) t_8/5_ along the line A in Figure 4.

Figure 8 shows the predicted thermal cycles at the five points highlighted in Figure 4, and the variations in the peak temperature and t_8/5_ along these points are shown in Figure 9. The region below −2.5 mm represents the BM, where phase transformation does not occur during welding because the peak temperature is less than Ac_1_. The region between −2.5 and −0.5 mm, where the peak temperature lies between Ac_1_ and the melting temperature, represents the HAZ. The region shallower than −0.5 mm represents the FZ, where the peak temperature exceeds the melting temperature. Figure 9b shows that t_8/5_ of the FZ and the HAZ adjacent to the FZ is short, while it becomes longer for regions away from the FZ. This is because heat transfer occurs more actively from the FZ and HAZ to the outside. The t_8/5_ throughout the welded joint is within 3 s, which is sufficient for martensitic transformation, provided the desired PAGS has been achieved.

Referring back to Figure 4, points 1, 2, 3, 4, and 5 relative to the weld center line were 0, −0.6, −1.4, −2.2, and −3.2 mm, respectively. The location of each point relative to the welded joint was determined according to the peak temperature. Point 1 belongs to the FZ; points 2, 3, and 4 belong to the HAZ; and point 5 belongs to the BM. Each point was analyzed comprehensively to investigate the characteristics of the welded joint, and it was compared to the experimental results obtained from the same location. Table 4 summarizes the peak temperature and t_8/5_ of each point. The peak temperature and t_8/5_ at point 1 were approximately 1870 °C and 1.45 s, respectively. The respective peak temperatures of points 2, 3, and 4 were 1485, 1090, and 785 °C, and their t_8/5_ values were 1.57, 1.78, and 2.62 s, respectively. The differences between the thermal cycle at points 2, 3, and 4 indicate that the phase transformation and fraction can vary from location to location within the HAZ. Finally, the peak temperature of point 5 was approximately 550 °C, which was below the Ac_1_ temperature, and no phase transformation was observed at this location during welding.

### 4.3. Prediction of PAGS and CCT Diagram

**Figure 10:**
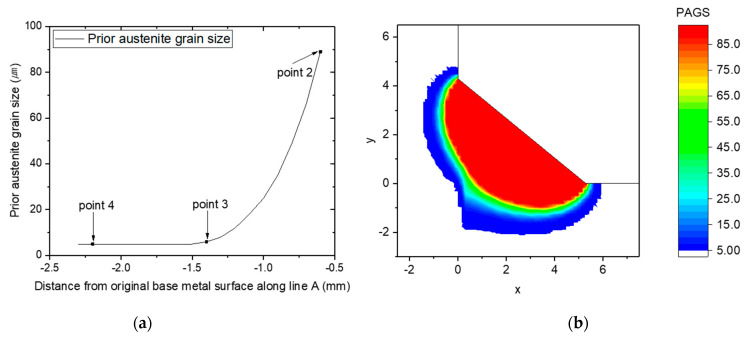
PAGS (**a**) along the line A in Figure 4 and (**b**) mapping of the cross-section.

A function of the PAGS with respect to the distance from the weld center line and the predicted PAGS mapping of the weld cross section is shown in Figure 10. Because austenite grain growth occurs in the temperature range between Ac_1_ and the melting temperature Tm, the PAGS was predicted only when the peak temperatures of points 2, 3, and 4 were within this temperature range.

Figure 10a shows that the PAGS increases insignificantly increases in the HAZ region (i.e., between −2.3 and −1.3 mm). The peak temperature in this range was more than 840 °C, which is higher than Ac_1_. In this range, the initial microstructure of the BM transformed into austenite; however, austenite grains did not grow because of the relatively low peak temperature and short residence time of the heat source. The PAGS started rapidly increasing at −1.3 mm, and the rate of growth increased as it approached the FZ; the peak temperature at this point was approximately 1130 °C. Locations closer to the FZ have a higher peak temperature above Ac_3_; therefore, coarse PAGS could form there. These results indicate that a range of PAGS exists in the welded joint and that its value increases while approaching the FZ. This demonstrates that multiple CCT diagrams are required to predict the phase fraction in a welded joint by accounting for various PAGS values.

The peak temperature at point 2 was approximately 1485 °C, which is significantly higher than Ac_3_, resulting in the complete transformation of the BM into austenite. The temperature and residence time at point 2 were high enough for austenite to grow, with the average grain size reaching approximately 90 μm. The peak temperature at point 3 was 1090 °C, causing complete conversion to austenite but no growth, owing to the relatively low peak temperature and short residence time above Ac_3_. This resulted in a relatively fine PAGS at point 3 at approximately 6 μm. Point 4 underwent a partial transformation to austenite because the peak temperature was 785 °C, which is between Ac_1_ and Ac_3_. Therefore, this section was not fully austenitized and retained some of the BM microstructure, and the austenite that formed had a grain size of approximately 5 μm. The peak temperature of point 1 was approximately 1870 °C, which is above the melting temperature. Therefore, the austenite grain size was not considered at point 1 because it was a region that existed in the liquid state during the welding process. Although the PAGS of the FZ was not considered, the PAGS used for the phase fraction prediction corresponded to the HAZ adjacent to the FZ. Therefore, Figure 10b appears to show the formation of PAGS of the FZ.

**Figure 11:**
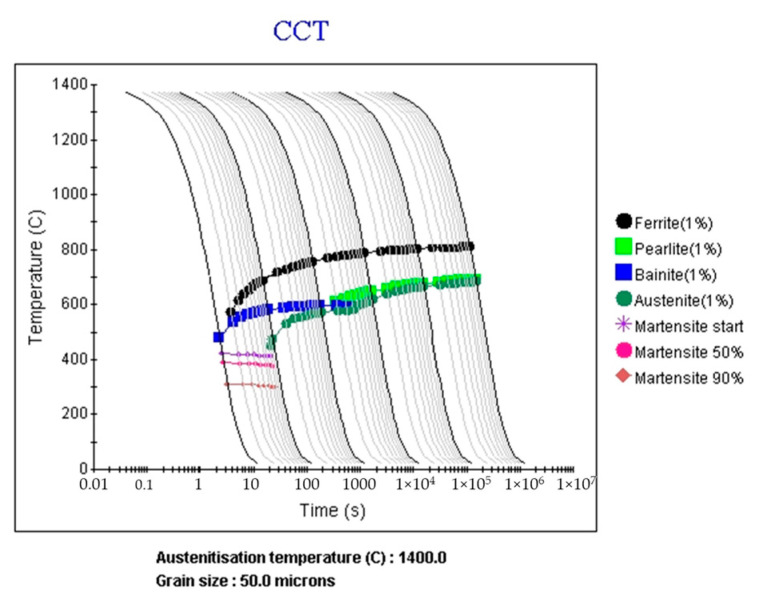
CCT diagram of SS400 steel at PAGS 50 μm.

Figure 11 shows the CCT diagrams with the composition of SS400 (Table 2) obtained for the PAGS of 50 μm that serve as the basis for the database established in Step 3. In this study, the CCT diagrams were calculated using JMatPro^®^13. 

**Figure 12:**
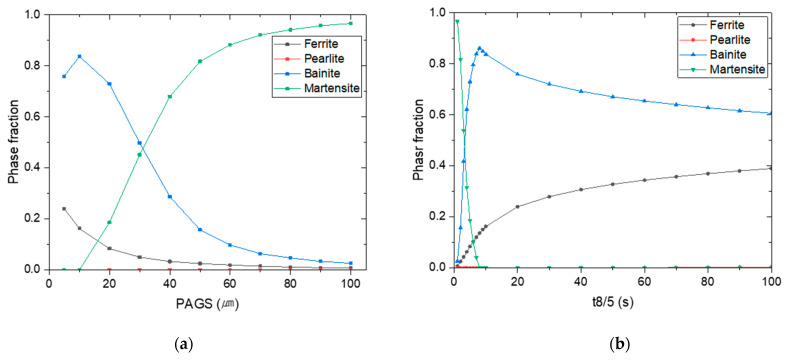
Phase fraction (**a**) according to PAGS when the t_8/5_ is 2 s and (**b**) according to t_8/5_ when PAGS is 50 μm.

Figure 12a and Table 5 show the phase fractions for various values of PAGS when t_8/5_ is 2 s. As shown in Figure 9b, t_8/5_ ranges from 1 to 3 s. Therefore, the effect of PAGS on the phase fraction was investigated when the median value of t_8/5_ was 2 s. The results indicate that larger PAGS promotes martensite transformation, while smaller PAGS results in bainite and ferrite transformation. Moreover, when PAGS is less than 10 μm, martensite is not formed even if t_8/5_ is short, which suggests that martensite transformation with a small PAGS may not occur even if the cooling rate is high. The reason for this is that when the PAGS is large, the grain boundary fraction is small, which provides a minimal number of nucleation sites for the ferrite transformation [38,39]. Hence, the transformation from austenite to martensite is favored over the transformation to ferrite and bainite. Conversely, a smaller PAGS enhances the transformation to ferrite, reducing the probability of martensite transformation. The phase fraction difference between PAGSs at the same t_8/5_ indicates that multiple CCT diagrams must be used to predict the phase fraction. Figure 12b shows the variation in the phase fractions with t_8/5_ when PAGS is 50 μm. Although t_8/5_ ranged for 3 s, the results from 1 to 100 s were analyzed to confirm the effect of t_8/5_ on the phase fraction. The results indicate that martensite transformation occurs for t_8/5_ up to 8 s, whereas transformation into ferrite and bainite occurs predominantly at longer t_8/5_. This implies that, even if PAGS is large, martensite transformation does not occur unless t_8/5_ is short enough. Therefore, both PAGS and t_8/5_ must be considered to predict the phase fraction of the welded joint.

### 4.4. Prediction of Phase Transformation and Fraction

As depicted in the flow chart in Figure 1, the phase fraction of the welded joint can be predicted using the peak temperature, PAGS, t_8/5_, and CCT. When the peak temperature is more than Ac_3_ and all existing phases are completely austenitized, the phase fraction can be predicted only using PAGS and t_8/5_. In contrast, when the peak temperature is between Ac_1_ and Ac_3_, the austenite fraction must first be evaluated, as phase transformation occurs only for the austenitized fraction.

**Figure 13:**
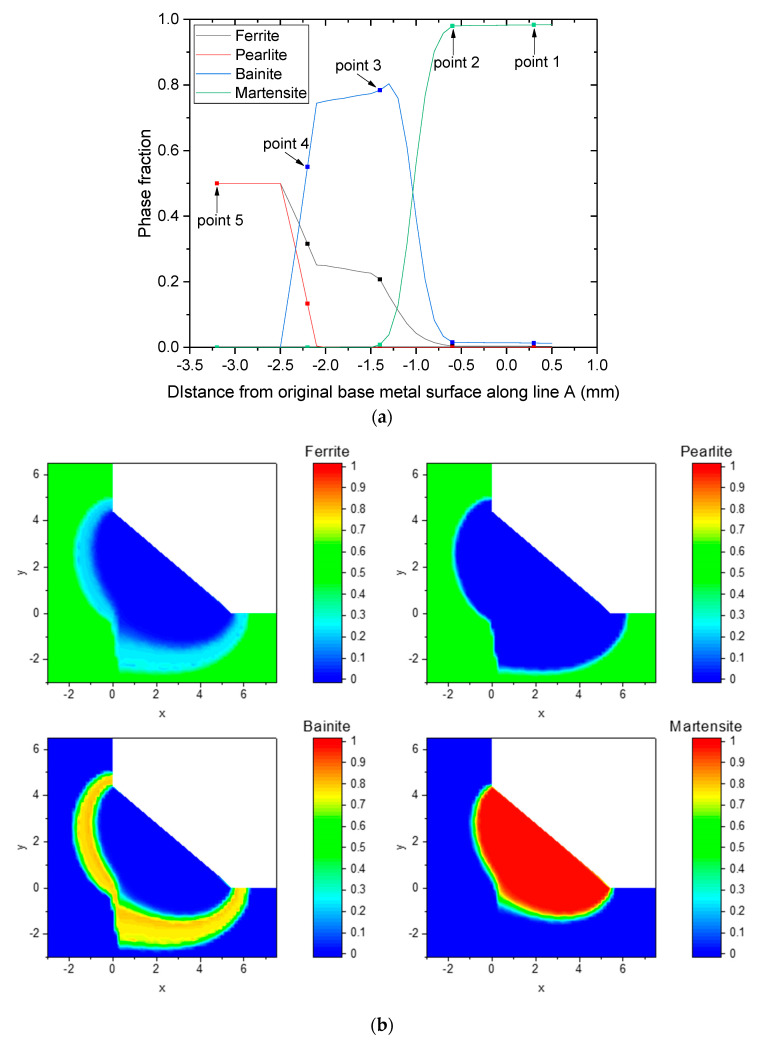
Phase fraction (**a**) along the line A in Figure 4 (**b**) mapping of the cross-section.

Figure 13a shows the predicted phase fraction at the points shown in Figure 4. Martensite is mainly observed to form at points 1 and 2 owing to the large PAGS and short t_8/5_. Point 3 consists of a large fraction of bainite and some ferrite owing to the relatively small PAGS. Point 4 includes bainite, ferrite, and pearlite, wherein the bainite decomposes from the partially austenitized phase, ferrite forms from both the partially dissolved austenitized phase and original non-austenitized BM, and pearlite remains in the original BM without being austenitized.

The same procedure can be applied to predict phase fractions for lines and planes (as seen in Figure 4). However, as shown in Figure 10, various distributions of PAGS are formed in the welded joint, which increases the effort required to predict the CCT diagram. Therefore, in this study, a database for phase fraction prediction with PAGS and t_8/5_ as variables was constructed based on the CCT diagram predicted according to the composition, as described in Section 4.1.

Variations in the phase fractions along line A depicted in Figure 4 are shown in Figure 13a, where the martensite fraction was observed to be the highest in the FZ and in the HAZ adjacent to the FZ, with a value close to 1. The high fraction of martensite in this region occurred because the martensite transformation from austenite was dominant owing to the coarse PAGS and high cooling rate. The fraction of martensite gradually decreased with PAGS, and the martensite fraction was negligible for distances less than −1.4 mm, as shown in Figure 13a. The bainite fraction was close to zero in the FZ and HAZ near the FZ owing to the large PAGS and high cooling rate. The bainite fraction was higher for low martensite fractions, and it gradually decreased upon approaching the BM. A small ferrite fraction was formed in the FZ and HAZ adjacent to the FZ, and the fraction increased toward the HAZ close to the BM, where the martensite and bainite fractions were low. As shown in Figure 13a, the ferrite fractions started to increase from −0.7 mm, where the martensite fraction and PAGS started to decrease. Therefore, the high fractions of bainite and ferrite were correlated with fine PAGS. Figure 9 shows that the peak temperature beyond −2.6 mm was below Ac_1_; hence, the area below −2.6 mm did not undergo a phase transformation during the welding process, and the untransformed BM microstructures remained. In this region, both the ferrite and pearlite fractions were assumed to be 0.5.

Figure 13b shows the distribution of each phase fraction across the cross section of the entire welded joint. Similarly to the result shown in Figure 13a, martensite was mainly distributed in the FZ and HAZ around the FZ. This result is comparable with the PAGS map shown in Figure 10, indicating that PAGS has a significant effect on martensite formation. The bainite first increased and then decreased with an increase in the distance from the FZ, and the ferrite gradually increased from the FZ to the BM. 

### 4.5. Microstructure and Grain Morphology of Experiments

**Figure 14:**
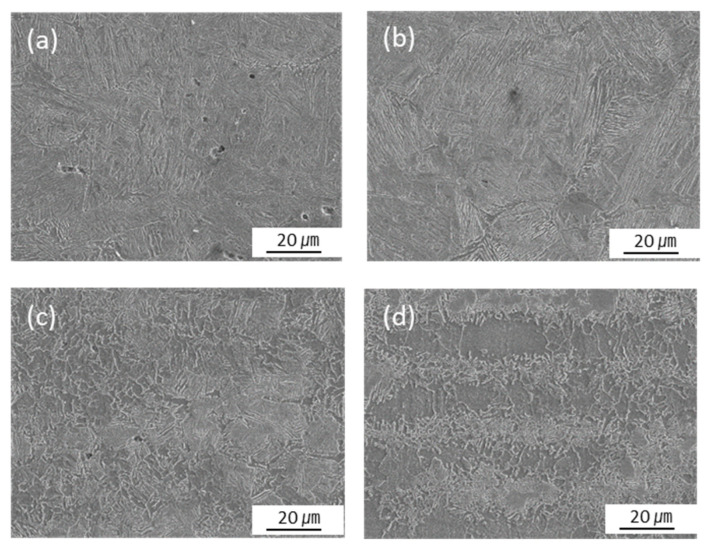
SEM image of microstructure in the welded joint: (**a**) point1, (**b**) point2, (**c**) point3 and (**d**) point4.

**Figure 15:**
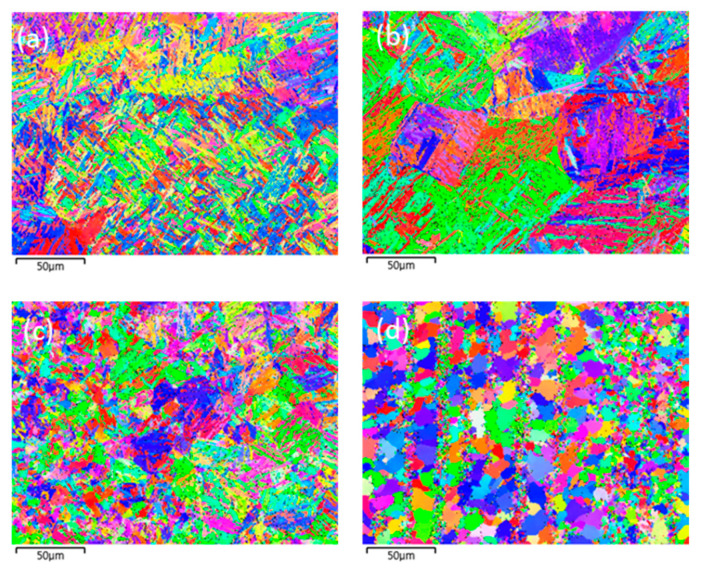
EBSD image of grain morphologies in the welded joint: (**a**) point1, (**b**) point2, (**c**) point3 and (**d**) point4.

The microstructures and grain morphologies at various locations in the welded joint are presented in Figure 14 and Figure 15. The locations chosen for Figure 14 and Figure 15 are those shown in Figure 4 and are the same where the phase fractions were predicted. As previously predicted using the model presented in this study, the microstructure of point 1 consists of martensite with an irregular lath phase, as shown in Figure 14a. Figure 14b and Figure 15b indicate that the microstructure of point 2 was predominately composed of lath martensite, and a large PAGS was observed. The microstructure of point 3 consisted of bainite and some ferrite, as shown in Figure 14c. Point 3 had a small PAGS, and the final grain morphology was fine, as shown in Figure 14c and Figure 15c. This was because the grain growth of austenite did not occur at point 3, owing to a low peak temperature and a short period of heating, as previously discussed. The microstructure of point 4 consisted of bainite, ferrite, and pearlite, as shown in Figure 14d. Here, pearlite and ferrite were the untransformed phases. Figure 15d shows a range of grain sizes at point 4 with some refined grains but no austenitized grains that retained the morphology of the BM.

### 4.6. Prediction of the Microhardness of Welded Joint and Comparison with Experimental Results

The microstructure has a significant effect on microhardness, which is closely related to other mechanical properties. In addition, the microhardness measurement provides a simple method for assessing the mechanical properties of a welded joint. The microhardness of the phases in order of highest to lowest is martensite, bainite, pearlite, and ferrite [9,40,41]. Quantitative analysis of microstructures via experiments is difficult; however, the predicted phase fraction can be verified by comparing the predicted microhardness with the experimentally obtained results. 

**Figure 16:**
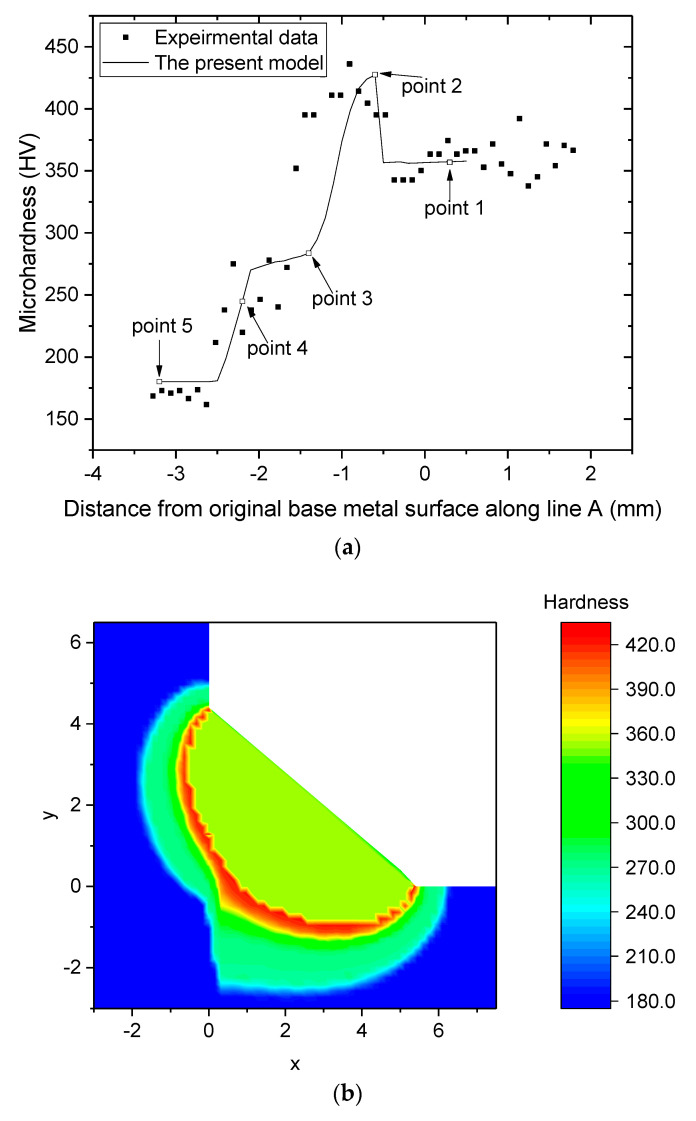
Microhardness (**a**) comparison between predicted and experimental results along the line A in Figure 4, and (**b**) mapping of the cross-section.

Figure 16 presents the experimental and predicted microhardness across 5 mm and the mapping results predicted using the phase-dependent microhardness. Figure 16a indicates that the predicted microhardness exhibited a trend similar to the measured results with no significant difference between the values. The biggest discrepancy is in the −1.1 to −1.5 mm range, where the peak temperature was 1050–1230 °C. In this range, the formation of fine grains was predicted owing to the small PAGS and high cooling rate; however, the effect of the grain size on the microhardness equation was not accurately considered. Figure 16a shows that the microhardness in the welded joint can be divided into regions where the values are increasing and regions where they are nearly constant. The region ranging from −2.5 to −3.2 mm consistently exhibits the lowest microhardness value, with an average value of 180 HV. This can be associated with a uniform microstructure of the BM, ferrite, and pearlite. Between −2.5 and −1.4 mm, the microhardness increases from approximately 180 to 280 HV. This increase coincides with the region where the microstructure of the BM was austenitized with minimal grain growth, as shown in Figure 10. This increase may be attributed to a decrease in the fraction of ferrite with a lower hardness and an increase in the fraction of bainite with a higher hardness as the distance from the BM increases, as shown in Figure 11. In this range, the absence of martensite limits the highest value of microhardness to 280 HV.

In the range from −1.4 to −0.6 mm, the microhardness increases from 280 HV up to 425 HV (the highest measured value). This increase may be attributed to the presence of martensite formed from the large PAGS and long t_8/5_. The microhardness then decreases to approximately 360 HV at −0.6 mm, where it exhibits relatively uniform values. This region is the boundary between the HAZ and FZ, which can be explained by the difference in their carbon contents. The microhardness of each phase increases with increasing carbon content, as shown by Equations (7)–(9), with martensite exhibiting a particularly significant increase in microhardness below 0.8% carbon content [42,43,44]. The higher carbon content in the HAZ (SS400 steel) compared to the FZ, which is a mixture of Er70s-6 and SS400 steel, results in higher microhardness values for each phase in the HAZ. As a result, although the phase fractions of the FZ and HAZ adjacent to the FZ are similar, their microhardness values differ by approximately 70 HV. The difference in microhardness between the FZ and the HAZ adjacent to the FZ can also be observed in the microhardness mapping result in Figure 16b.

## 5. Conclusions

This study proposes a methodology to predict phase fractions and microhardness of an entire welded joint and analysis of the phase transformation during welding. The methodology combines a three-dimensional numerical heat transfer model and procedure for predicting the phase fractions of a welded joint. The heat transfer model was used to simulate the heat transferred in the welded joint and obtain thermal cycles, and the procedure involved obtaining the peak temperature, austenite fraction, PAGS, and t_8/5._ The prediction of the austenite fraction was based on the peak temperature, while the PAGS was predicted using a grain growth model, and the phase fraction was then predicted using a database created based on CCT diagrams. The reliability of the heat transfer model was verified by comparing the weld bead geometry and thermal cycles with the experimental results, and the predicted phase fraction and microhardness were verified by comparing them with the experimental results measured along points and lines (as seen in Figure 4). The predicted phase fractions and microhardness were found to be in agreement with experimental results, demonstrating the accuracy of the methodology. 

The PAGS in HAZ increased toward the FZ, owing to a high peak temperature and the increase in the residence time above the Ac_3_ temperature. The martensite fraction was highest in the HAZ adjacent to the FZ and gradually decreased with increasing distance from the FZ. Bainite formed from the middle of the HAZ, and its fraction decreased as the distance from the BM decreased. Ferrite was present throughout the welded joint, and its fraction gradually increased toward the BM with a small PAGS. The predicted microhardness values also show that the maximum microhardness occurred in the HAZ near the fusion line, owing to the high fraction of martensite and a high carbon concentration. The microhardness gradually decreased toward the BM, primarily owing to a lower martensite fraction and a higher ferrite fraction.

The methodology presented in this study will be valuable in analyzing phase transformation and fraction according to various welding conditions, which are difficult to perform owing to experimental limitations. Although the phase fraction and microhardness were predicted for GTAW and SS400 steels in this study, the methodology can be applied to other welding methods and materials given the thermal cycles and a database based on CCT diagrams. In future studies, the methodology could be applied to SAW and used in multilayer welding, which has been difficult to investigate previously because of the formation of microstructures more complex and diverse than those produced via single-pass welding. 

## Figures and Tables

**Table 1:** Submerged arc welding (SAW) and gas tungsten arc welding (GTAW) parameters used for the heat transfer model.

Property	Case 1: SAW		Case 2: GTAW
	Internal Pass	External Pass	Single Pass
Density (kg/m^3^)	7500		Temperature-dependent function
Specific heat [J/(kg·K)]	Temperature-dependent function		726 (solid), 732 (liquid)
Thermal conductivity [W/m·K)]	Temperature-dependent function		Temperature-dependent function
Viscosity [kg/(m·s)]	Temperature-dependent function		Temperature-dependent function
Pure solvent melting heat (J/kg)	270,000		270,000
External emissivity	0.4		0.4
Flux thickness (mm)	20		-

**Table 2:** Chemical composition of the base metal and filler metal for SAW and GTAW.

Process	Material	C	Si	Mn	Ni	Cr	Mo	Fe
Case 1: SAW	ASTM A516-60 (Base metal)	0.158	0.227	0.95	0.012	0.341	0.0018	Bal.
	API 2H-50 (Filler metal)	0.124	0.202	1.512	0.305	0.024	0.068	Bal
Case 2: GTAW	SS400 (Base metal)	0.17	0.21	1.06	0.005	0.02	0.02	Bal.
	Er70s-6 (Filler metal)	0.071	0.84	1.50	0.01	0.03	0.001	Bal.

**Table 3:** Conditions of welding process for SAW and GTAW.

Process		Voltage (V)/Current (A)	Weld Velocity (mm/s)	Heat Input (kJ/mm)
Case 1: SAW	Internal	DC: 31/620 AC1: 33/560, 34/520	21.6	2.56
	External	DC: 32/830 AC1: 35/520, 35/500		2.87
Case 2: GTAW		18.5/160	1.92	1.54

**Table 4:** Peak temperature, t_8/5_ and PAGS at different locations.

	Distance from Weld Center Line (mm)	Peak Temperature (°C)	T_8/5_ (s)	PAGS (μm)
Point 1	0	1872	1.45	-
Point 2	−0.6	1485	1.57	103
Point 3	−1.4	1090	1.78	6.4
Point 4	−2.2	785	2.62	5
Point 5	−3.2	551	-	-

**Table 5:** Phase fraction according to PAGS when the t_8/5_ is 2 s.

PAGS (μm)	Ferrite	Pearlite	Bainite	Martensite
5	0.24	0.00	0.76	0.00
10	0.16	0.00	0.84	0.00
20	0.08	0.00	0.73	0.19
30	0.05	0.00	0.50	0.45
40	0.03	0.00	0.29	0.68
50	0.02	0.00	0.16	0.82
60	0.02	0.00	0.10	0.88
70	0.02	0.00	0.06	0.92
80	0.01	0.00	0.05	0.94
90	0.01	0.00	0.03	0.96
100	0.01	0.00	0.02	0.97

## Data Availability

Not applicable.

## Nomenclature

Ac_1_	austenite start temperature
Ac_3_	austenite complete temperature
$C_{p}$	specific heat capacity
*D*	final grain size
*D*_0_	initial grain size
$\vec{F}$	surface tension
$\vec{g}$	gravitational acceleration
*h*	sensible enthalpy
*H*	enthalpy
*k*	thermal conductivity
*K*	kinetic constant
*L*	latent heat
*n*	time exponent
*P*	pressure
*Q* *_app_*	activation energy for the grain boundary movement
*t*	time
*t*_1_, *t*_2_	grain growth start, end times
*T*	temperature
*T*(*t*)	the time-dependent thermal cycle
Tm	melting temperature
$T_{liquidus},T_{solidus}$	liquidus and solidus temperature
t_8/5_	the cooling time between 800 and 500 °C
$\vec{v}$	fluid velocity
V	cooling rate at 700 °C
*Greek symbols*	
β	liquid fraction
ρ	density
μ	viscosity
